# Thyroid function and associated mood changes after COVID-19 vaccines in patients with Hashimoto thyroiditis

**DOI:** 10.3389/fimmu.2023.1129746

**Published:** 2023-04-06

**Authors:** Yifei Ma, Jiling Zeng, Yongluo Jiang, Yi-Wei Xu, Youlong Wang, Guanqing Zhong, Nianqi Liu, Yanqi Wang, Zhiying Zhang, Yiming Li, Shuqin Chen, Xiao-Long Wei, Pengfei Zhu, Guangmin Jian, Xiajie Lyu, Yu Si Niu, Mingwei Li, Shuang Liang, Guangzhen Fu, Shaohui He, CanTong Liu, Ao Zhang, Xinjia Wang

**Affiliations:** ^1^ Orthopedics and Spine Surgery, Second Affiliated Hospital of Shantou University Medical College, Shantou, Guangdong, China; ^2^ Nuclear Medicine, State Key Laboratory of Oncology in South China, Collaborative Innovation Center for Cancer Medicine, Sun Yat-sen University Cancer Center (SYSUCC), Guangzhou, Guangdong, China; ^3^ Clinical Laboratory Medicine, Cancer Hospital of Shantou University Medical College, Shantou, China; ^4^ General Surgery, Hainan Branch of People’s Liberation Army General Hospital, Sanya, Hainan, China; ^5^ Department of Clinical Laboratory, State Key Laboratory of Oncology in South China, Guangdong Key Laboratory of Nasopharyngeal Carcinoma Diagnosis and Therapy, Sun Yat-sen University Cancer Center, Guangzhou, China; ^6^ Faculty of Psychology, Institute of Educational Science, Huazhong University of Science and Technology, Wuhan, Hubei, China; ^7^ Bone and Soft Tissue Oncology, Cancer Hospital of Shantou University Medical College, Shantou, Guangdong, China; ^8^ School of Public Health, Shantou University, Shantou, Guangdong, China; ^9^ Bone and Soft Tissue Oncology, Guangdong Provincial Key Laboratory for Breast Cancer Diagnosis and Treatment, Cancer Hospital of Shantou University Medical College, Shantou, Guangdong, China; ^10^ Neurosurgery, Beijing Tiantan Hospital, Capital Medical University, Beijing, China; ^11^ Pathology, Cancer Hospital of Shantou University Medical College, Shantou, Guangdong, China; ^12^ Clinical Laboratory, First Affiliated Hospital of Zhengzhou University, Zhengzhou, Henan, China; ^13^ Environmental Health, Rollins School of Public Health, Emory University, Atlanta, GA, United States; ^14^ Acute Communicable Disease Epidemiology Division, Dallas County Health and Human Services, Dallas, TX, United States; ^15^ Pediatric Dentistry, The Stomatological Hospital Affiliated to medical school of Nanjing university, Nanjing, China; ^16^ Teaching Department for Students with Cerebral Palsy, Shanghai Pudong New District Special Education School, Shanghai, China; ^17^ Clinical Laboratory, Key Clinical Laboratory of Henan Province, First Affiliated Hospital of Zhengzhou University, Zhengzhou, Henan, China; ^18^ Orthopaedic Oncology, No.905 Hospital of People's Liberation Army (PLA) Navy, Changzheng Hospital, Naval Medical University, Shanghai, China

**Keywords:** COVID-19 vaccines, Hashimoto thyroiditis, thyroid function, inflammation, mood change

## Abstract

**Context:**

Severe acute respiratory syndrome-coronavirus 2 (COVID-19) vaccines may incur changes in thyroid functions followed by mood changes, and patients with Hashimoto thyroiditis (HT) were suggested to bear a higher risk.

**Objectives:**

We primarily aim to find whether COVID-19 vaccination could induce potential subsequent thyroid function and mood changes. The secondary aim was to find inflammatory biomarkers associated with risk.

**Methods:**

The retrospective, multi-center study recruited patients with HT receiving COVID-19–inactivated vaccines. C-reactive proteins (CRPs), thyroid-stimulating hormones (TSHs), and mood changes were studied before and after vaccination during a follow-up of a 6-month period. Independent association was investigated between incidence of mood state, thyroid functions, and inflammatory markers. Propensity score–matched comparisons between the vaccine and control groups were carried out to investigate the difference.

**Results:**

Final analysis included 2,765 patients with HT in the vaccine group and 1,288 patients in the control group. In the matched analysis, TSH increase and mood change incidence were both significantly higher in the vaccine group (11.9% versus 6.1% for TSH increase and 12.7% versus 8.4% for mood change incidence). An increase in CRP was associated with mood change (p< 0.01 by the Kaplan–Meier method) and severity (r = 0.75) after vaccination. Baseline CRP, TSH, and antibodies of thyroid peroxidase (anti-TPO) were found to predict incidence of mood changes.

**Conclusion:**

COVID-19 vaccination seemed to induce increased levels and incidence of TSH surge followed by mood changes in patients with HT. Higher levels of pre-vaccine serum TSH, CRP, and anti-TPO values were associated with higher incidence in the early post-vaccine phase.

## Introduction

Hashimoto thyroiditis (HT) is an autoimmune condition featured by lymphocytic destruction and chronic inflammation of thyroid gland (1, 2), and pathological process involves the interaction between the genetic and environmental factors (2). Antibodies of thyroid peroxidase (anti-TPO) and/or thyroglobulin (anti-TG) are increased, which serve as the main criteria for diagnosis if combined with symptoms of hypothyroidism or low echogenicity in ultrasound. HT is usually underdiagnosed because of symptomatic dormancy. Foreign antigen–associated immune alterations, followed by internal inflammation, were hypothesized as one of the probable reasons for symptomatic conversion from long-term dormancy (3–5).

In the era of COVID-19 posing the greatest threat to public health, the introduction of various types of vaccines successfully decreased the rate of COVID-19 infection for both healthy populations and those with underlying diseases (6, 7). Immune response and safety in patients with HT have been concretely evidenced, and all patients are recommended to receive vaccines (8, 9). To date, sparse cases of thyroiditis in the general population have been reported after vaccination, and thyroid function changes are reported in healthy subjects recently (10–13). However, inflammatory and corresponding phenotypical changes have not been investigated after the introduction of a foreign antigen into immune system in the current stage when all patients are suggested to receive COVID-19 vaccines.

Next, whether vaccine-associated inflammatory marker changes are inducible to thyroid dysfunction and symptom changes in patients with HT has not been studied. Thyroid functions are critically linked to mood states, and the rate of mood swings is significantly higher in the HT population, with reports in the large samples of three times of incidence in 1 year as compared to general population (14, 15). Considering the association with antigen presentation and subsequent immune upregulation, changes in disease status are possible from subclinical to apparent symptoms. Incidence of mood state disorders in the era of COVID-19 pandemic requires further investigation due to the high levels of stress-afflicted HT people, which represents a psychiatrically susceptible group of patients with potential immune and thyroid dysregulation (11, 16). Such investigation may offer an added value to clinicians and patients who demand data on long-term influence of vaccines on subclinical HT. Thus, in the current study, we aim to find the vaccination-associated thyroid function and mood changes and to investigate how related inflammatory markers influence the outcomes.

## Methods

### Patients and study design

The retrospective, multi-center study recruited patients with clinical or subclinical HT to receive standard two-dose inactivated CoronaVac (BBIBP-CorV) vaccines during May 2021 to January 2022. Because there have not been guidelines for vaccine safety in China for patients with HT during recruitment period, vaccination was primarily decided voluntarily by patients. Thus, patients not receiving vaccines during study period were included as the control group, and corresponding matching by propensity scores was done to reduce bias. Diagnosis of HT was the presence of clinical symptoms of hypothyroidism combined with the increased levels of anti-TPO and/or anti-TG antibodies. Ethical approval was obtained at the Second Affiliated Hospital of Shantou University Medical College as a waiver option for the retrospective study protocol. This study was performed according to the principles of Declaration of Helsinki, Good Clinical Practice, and all patients provided informed consent before participation.

The primary outcome was to evaluate the mood and thyroid function changes after COVID-19 vaccination, and an exploratory analysis was carried out to identify potential associations between inflammation markers and primary outcome. To illustrate role of vaccines and to control for the potential psychiatric-related conditions during COVID-19 pandemic, a control cohort with HT was recruited in the same hospitals that did not receive vaccines as geographical match during study period. The smallest sample size was calculated by PASS (V 15.0) before recruitment to reach the effect size of the thyroid-stimulating hormone (TSH) levels of 5% difference with pre-specified alpha of 0.05 and beta of 0.20, and the upper limit was dependent upon the actual size of recruited during study period (consecutive recruitment). Key exclusion criteria of the study are as follows: 1) critically ill conditions with a survival prognosis of less than 1 year; 2) diagnosis of Grave’s disease; 3) history of anaphylaxis to contents of vaccine products; and 4) acutely ill patients or patients with active cancers, who may show increased inflammation markers.

### Baseline and follow-up assessment

The demographic data were extracted from Case Record Forms of the five tertiary referral hospitals, and the electronic data capture systems were applied to save and monitor de-identified profiles, in which each patient was coded for follow-up track. Demographic information included gender and age. Patients were followed up online to test their mood state on a weekly basis, and serum tests were performed in clinical laboratories of enrollment centers. Follow-up started from the date of vaccination to the 24th week after vaccination (end of follow-up).

The serum levels of markers were tested one to three times during a 3-month period prior to vaccination and at the end of follow-up. Mean values were applied in final calculation to represent baseline inflammation profiles. Markers included C-reactive protein (CRP, μg/dl; 1 mg/L = 100 μg/dl), interleukin-6 (IL-6), TSHs, and anti-TPO and anti-TG antibodies. At the full length of follow-up, Beck Depression Inventory (BDI) was administered online at each weekend to assess the mood change levels of patients dating back 2 weeks. Definition of mood changes was a BDI score of more than or equal to 13 (17, 18). Diagnosis of mood changes was further validated by symptoms during past 2 weeks in clinical settings at community or referral psychiatry clinics if the patient scored more than 13. Patients with suicidal or aggressive symptoms, if any, were medically managed and properly recorded.

Clinic coordinators with supervision gather data in community clinics when patients were not reachable in the tertiary hospitals (either as inpatients or outpatients). All data were extracted from Case Record Form of the follow-up sites, and the electronic data capture systems were applied to save and monitor de-identified profiles.

### Multivariate regression, survival analysis, and nomogram development

Incidence rate of mood change during follow-up was determined with the Kaplan–Meier method. In both vaccine and control groups, the incidence curves were drawn, and the log-rank test was applied to compare difference between different groups. Markers significant in univariate survival analysis were subject to multivariate Cox proportional hazards models to identify independent pre-vaccination markers that predict outcomes, with the corresponding hazard ratio and 95% confidence intervals. Two nomograms were formulated by using package of rms in R version 4.0.5 (http://www.r-project.org/). First, nomogram was constructed to predict incidence of TSH increase and, second, was built to predict disease-free survival (DFS) time during follow-up (19). The performance of nomograms was measured by concordance index (C-index) and by comparing the nomogram-predicted versus observed rates of events (i.e., DFS and TSH increase). Bootstraps with 1,000 resampling were used.

### Propensity score–based outcome analysis between the vaccine and control groups

To further decrease confounding bias across chronologically and geographically different enrollment and to alleviate pandemic-related psychiatric confounders, propensity score–based matching was performed between the vaccine and control groups. Propensity scores were calculated with the logistic conditional regression models. Variables included were those significantly associated with incidence of mood change and/or the increase of TSH levels in multivariate regression and survival analysis (20). Nearest neighbor head-to-head (1:1) method was adopted to match each participant in the vaccine and control groups, respectively, with a caliper width of 0.2 without replacement (20). Standardized mean difference (SMD) was calculated and compared between unmatched and matched data to evaluate matching performance as imbalance test. An SMD over (√((n1+n2)/(n1*n2)))*1.96 was regarded as an imbalanced test result (20). Statistical tests of difference in matched samples included McNemar tests for categorical variables and Wilcoxon signed-rank tests for continuous variables (20).

### Statistic calculation

The categorical factors were represented as numbers and percentages, and the continuous factors were represented as means ± standard deviations and median (25th to 75th quartile). Each statistical test was based on a pre-determined statistical hypothesis with a type I error of 0.05. The paired Student’s t-test was applied to compare continuous variables of the same cohort in chronological settings for parametric tests, including changes in CRP, BDI scores, and TSH values. Statistics used in comparison study were carried out in SPSS V.24.0 software.

## Results

### Patient characteristics

The study enrolled 4,556 patients with HT, and 503 patients did not consent to follow-up and thus were excluded. Thus, final analysis included 2,765 patients in the vaccine group and 1,288 patients in the control group (3,198 female patients and 855 male patients; mean age, 42.34 ± 14.99 years). Before vaccination, mean CRP levels of the vaccine group were 581.12 ± 789.94 μg/dl, and mean TSH values were 293.29 ± 111.70 μIU/dl. Anti-TG antibody levels were 92.17 ± 375.92 IU/ml, and BDI scores were 8.52 ± 5.17. Anti-TPO antibody levels were 38.36 ± 100.55 IU/ml. Among all participants, 499 patients had a prior history of psychiatric diseases, and 104 patients had other autoimmune diseases (AIDs). Baseline demographics and serum markers of both groups are shown in **Table 1**.

**Table 1:** Baseline variables of patients with Hashimoto thyroiditis.

		Before matching			After matching (N = 1,039 pairs)		
Variables		Vaccine group N = 2,765	Control N = 1,288	*P* ^1^	Vaccine group	Control	*P* ^2^
Age (years)	Mean (SD)	42.34 (13.99)	51.84 (14.91)	<0.01	47.39 (12.78)	48.69 (14.01)	0.03
	Median (quartile)	41 (31–54)	52 (42–63)		48 (37–56)	50 (39–58)	
Gender	Male	415 (15.0)	440 (34.2)	<0.01	286 (27.5)	261 (25.1)	0.21
	Female	2350 (85.0)	848 (65.8)		753 (72.4)	778 (74.9)	
Time since diagnosis (months)	Mean (SD)	9.60 (10.95)	10.14 (6.65)	0.05	9.40 (8.87)	9.74 (6.64)	0.33
	Median (quartile)	5 (3–10)	9 (5–14)		6 (4–10)	8 (4–14)	
TSH (μIU/dl)	Mean (SD)	293.29 (111.70)	292.60 (119.35)	0.86	291.13 (108.37)	292.14 (119.30)	0.84
	Median (quartile)	291 (201–384)	292 (189–393)		290 (204–378)	293 (189–394)	
CRP (μg/dl)	Mean (SD)	581.12 (789.94)	435.02 (476.71)	<0.01	487.52 (684.91)	447.42 (489.24)	0.13
	Median (quartile)	190 (80–740)	240 (130–650)		180 (70–620)	240 (140–180)	
IL-6 (pg/ml)	Mean (SD)	146.97 (103.33)	–	–	148.52 (105.02)	–	–
	Median (quartile)	117 (64–204)	–		118 (64–207)	–	
Anti-TG (IU/ml)	Mean (SD)	92.17 (375.92)	–	–	117.62 (448.82)	–	–
	Median (quartile)	15.81 (13.91–33.83)	–		16.14 (13.97–14.03)	–	
Anti-TPO (IU/ml)	Mean (SD)	38.36 (100.55)	56.82 (121.81)	<0.01	48.30 (121.10)	50.92 (112.68)	0.61
	Median (quartile)	11.37 (8.67–17.31)	14 (8–19)		11.48 (8.73–18.94)	14 (8–19)	
Comorbidity with other AIIDs	Yes	2661 (96.2)	1168 (90.7)	<0.01	85 (8.1)	68 (6.5)	0.15
	No	104 (3.8)	120 (9.3)		954 (91.9)	971 (93.5)	
BDI values	Mean (SD)	8.52 (5.17)	8.29 (4.40)	0.08	8.91 (5.35)	8.26 (4.41)	0.03
	Median (quartile)	8 (4–12)	8 (5–12)		9 (4–12)	8 (5–12)	
Psychiatric disease history	No	2266 (82.0)	984 (76.4)	<0.01	806 (77.6)	806 (77.6)	1.00
	Yes	499 (18.0)	304 (23.6)		233 (22.4)	233 (22.4)	

SD, standard deviation; AIIDs, autoimmune inflammatory diseases; BDI-13, Beck Depression Inventory; TSH, thyroid stimulating hormones; CRP, C-reactive proteins.

**^1^** Independent t-test (continuous variable) and chi-square test (categorical variable).

**^2^** Wilcoxon signed-rank test (continuous variable) and McNemar test (categorical variable).

### Thyroid function and mood changes after COVID-19 vaccination

As there could be episodes of incident mood and thyroid function changes during course of HT that were potentially unrelated to vaccination, baseline and follow-up data of the control group were compared with those of the vaccine group after propensity score matching to find whether incident mood changes were associated with vaccination. Matching yielded a total of 1,039 pairs, with the imbalance test results shown in **Supplementary Table 1**. Bias-corrected total incidence of mood changes during follow-up was 12.7% in the vaccine group and 8.4% in the control group. Kaplan–Meier survival analysis between the two groups showed that there was a significant difference between the two groups in incidence of mood changes (**Supplementary Figure 1**).

**Figure 1:**
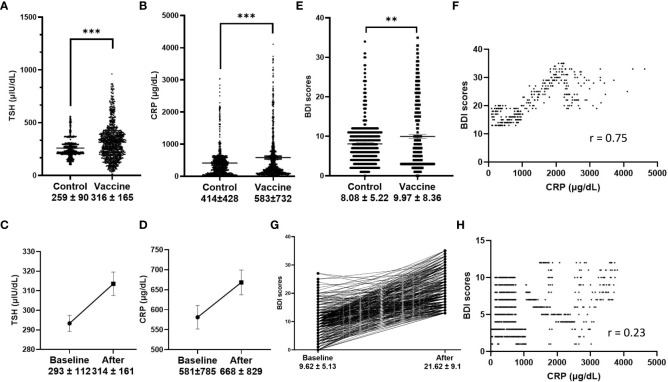
Serum markers and Beck Depression Index (BDI) scores. **(A, B)**, TSH and CRP values in the vaccine group and control group (p < 0.001 in matched analysis); **(C, D)**, TSH and CRP values before and after vaccination (p < 0.001); **(E)**, BDI scores in the vaccine group and control group, (p < 0.001 in matched analysis); **(F)**, CRP values were correlated with BDI scores after vaccination in the subgroup of patients with greater mood changes (defined as BDI score > 13, r = 0.75), and no correlation was found in subgroup without greater mood changes **(H)**; Changes of BDI scores before and after vaccination in the subgroup with greater mood changes **(G)**.

Using the McNemar test, the percentage rate of TSH increase was significantly different between the two groups (11.9% versus 6.1% for TSH increase in the vaccine and control groups, respectively). Further comparison of mean TSH values also showed significance (316 ± 195 μIU/dl versus 259 ± 90 μIU/dl, p< 0.01 by the Wilcoxon signed-rank test, **Figure 1A**). Another significant finding was the difference in the CRP values in the vaccine and control groups (583 ± 732 μg/dl versus 414 ± 428 μg/dl, p< 0.01 by Wilcoxon signed-rank test, **Figure 1B**). Changes in TSH and CRP values were also seen in the original, unmatched vaccine group before and after vaccination, respectively (p< 0.01, **Figures 1C, D**). To test the relationship between mood change severity and the level of CRP in the vaccine group, BDI scores were found to be correlated with the CRP levels during follow-up in the subgroup with mood changes (r = 0.75), and correlation was not found in the subgroup without mood changes (**Figures 1E–H**).

### Serum markers may independently predict incident mood change

**Figure 2:**
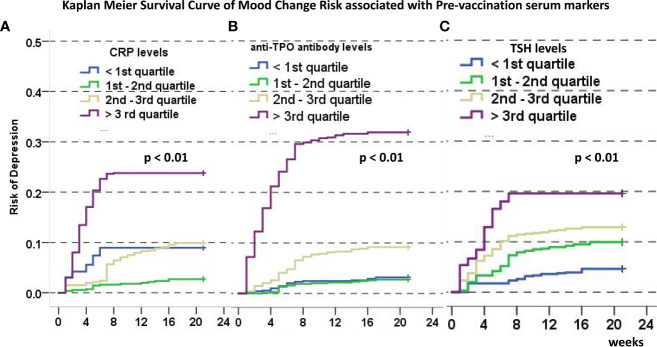
Kaplan-Meier Curve of disease-free survival (defined as time to mood changes) associated with CRP **(A)**, anti-TPO **(B)**, and TSH **(C)** levels categorized by the 25th, 50th, and 75th quartile. In all images, a higher ladder of marker categories predict higher incidence of disease-free survival.

**Figure 3:**
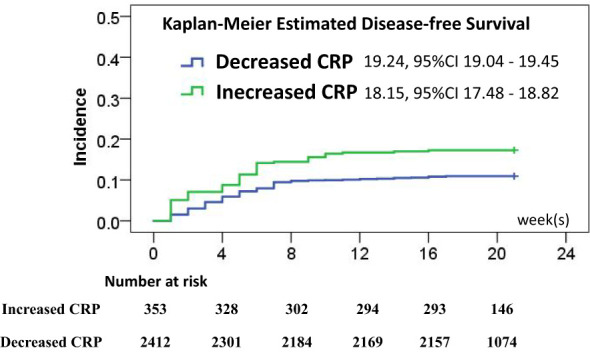
Disease-free survival rate estimates after vaccination associated with CRP changes, and the risk of mood changes was significantly different (p < 0.01).

To test the predicting values of serum markers before and after vaccination and to evaluate the relationship between demographics and rates of mood changes, univariate and multivariate survival analyses were carried out to find the independent markers associated with incidence of mood changes (**Table 2**). Initial Kaplan–Meier methods found that the baseline TSH values, CRP levels, IL-16 levels, and anti-TG antibody levels were associated with incidence of mood changes during follow-up. All variables but IL-6 levels were significant in multivariate survival analysis (p< 0.01). The Kaplan–Meier survival curves of CRP, TSH, and anti-TPO levels are shown in **Figure 2**. Incidence rate of mood changes was 17.3% in patients with increased CRP and 10.9% in patients without an increase. CRP increase after vaccination was associated with the increased mood change incidence (**Figure 3**).

**Table 2:** Survival analysis of incident mood changes after vaccination.

Variables		*P* (univariate)	*P* (multivariate)	HR (95% CI)
**Gender**		0.56	–	–
**Age**		0.27	–	–
**Time since diagnosis**		0.96	–	–
**TSH (μIU/dl)**	**<201**	<0.01	<0.01	0.27 (0.18–0.40)
	**201**–**291**	<0.01	<0.01	0.50 (0.38–0.67)
	**291**–**384**	<0.01	<0.01	0.71 (0.54–0.93)
	**>384**	Reference	Reference	Reference
**CRP (μg/dl)**	**<80**	<0.01	<0.01	0.36 (0.27–0.48)
	**80**–**190**	<0.01	<0.01	0.10 (0.06–0.17)
	**190**–**740**	<0.01	<0.01	0.56 (0.43–0.74)
	**>740**	Reference	Reference	Reference
**IL-6 (pg/ml)**	**<64**	<0.01	0.85	0.97 (0.69–1.36)
	**64**–**117**	<0.01	0.84	1.03 (0.75–1.42)
	**117**–**204**	<0.01	0.32	1.17 (0.86–1.58)
	**>204**	Reference	Reference	Reference
**Anti-TG antibody (IU/ml)**		0.67	–	–
**Anti-TPO antibody (IU/ml)**	**<8.67**	<0.01	<0.01	0.09 (0.06–0.15)
	**8.67**–**11.37**	<0.01	<0.01	0.08 (0.05–0.13)
	**11.37**–**17.31**	<0.01	<0.01	0.28 (0.21–0.37)
	**>17.31**	Reference	Reference	Reference
**Comorbidity with other AIIDs**		0.22	–	–
**BDI values**		0.55	–	–
**Psychiatric disease history**		0.31	–	–

AIIDs, autoimmune inflammatory diseases, BDI-13, Beck Depression Inventory, TSH, thyroid stimulating hormones; CRP, C-reactive proteins; HR, hazard ratio; CI, confidence interval.

### Nomogram predicting thyroid function changes and early-onset mood change incidence

Final analysis was done to see whether there was an association between mood change–predicting serum markers with change in thyroid function after vaccination. Baseline demographic and serum markers were subject to univariate and multivariate regression analyses of TSH increases in the vaccine group, and results are shown in **Supplementary Table 2**. It was demonstrated that the same markers were associated with TSH changes after vaccination. Thus, nomograms were developed in the vaccine group to quantify the risk of mood changes and the risk of TSH increases by means of anti-TPO antibodies, TSH, and CRP values before vaccination. In nomogram to predict TSH increases (**Supplementary Figure 2**), the value of C-index was 0.69, and the calibrating curve demonstrated a relatively good contingency between the predicted and actual rates of TSH increases.

## Discussion

As COVID-19 vaccines became widely covered in general population, thyroid function changes became evident in a small range of samples characterized by subclinical to clinical thyroiditis (12, 21). Thyroid function fluctuations in HT, autoimmune inflammatory thyroiditis (AIT), and mood state changes that ensued have seldom been reported. This study offered first observatory, real-world evidence that COVID-19 vaccination could incite changes in mood changes levels and thyroid function in HT by means of matched analysis. Specifically, associations between the increased CRP and incidence and the severity of mood changes were investigated in a relatively large sample.

Mood changes, a mood disorder that affects 6% to 7% of the general population, is quite commonly seen in patients with hypothyroid states due to the deranged levels of thyroid hormones and persistent inflammation (15, 22). Autoimmune thyroiditis was reported to be the most prevalent pathology type in such a population, and, by meta-analysis, 1-year prevalence of mood changes could be as high as 17% (14). The inflammatory state, represented by CRP levels and being a major trigger of mood swings in AIT, could be subject to fluctuations by means of an external stimulus such as COVID-19 vaccine antigens (3). Antigen-related CRP level increases were seen after vaccination (from 581 to 688 μg/dl), and the result was validated by comparison with the control group. Similar results have been reported in patients with psychiatric conditions, and an earlier study on influenza vaccination indicated changes in the inflammation levels (23, 24). Consistent with prior research, higher baseline CRP levels predict a higher probability of mood changes after immune stimulus, and, combined with hypothyroid states, an even higher risk could be predicted in current nomogram.

Safety of COVID-19 vaccines has been widely evidenced in recent studies of autoimmune conditions, and most effort has been intensified on immunogenicity and vaccine-related adverse events following vaccination (11). Reports on immune-related events after COVID-19 vaccination in autoimmune patients mainly focused on the immediate phenotypes in the small samples, and few studies gave real-world evidence on changes of inflammatory or hormone changes of AID, which were shown to predict future outcomes in a number of chronic conditions (25, 26). This work demonstrated that TSH values were increased after vaccination, and independent analysis found associations between CRP values and incidence of TSH increase after vaccination. Moreover, the same serum markers were shown associated with incidence and severity of mood changes. Considering prior research, clinicians are suggested to monitor potential changes in disease progression after COVID-19 vaccination. Future research is encouraged to find molecular mechanisms of vaccine-induced hormone and mood changes.

Our work bears several limitations. Follow-up time was relatively short such that long-term disease-related outcomes cannot be assessed. In addition, third-dose vaccines took place 6 months after the second dose, and we did not assess outcomes after booster vaccination, which may provide more data on serum marker changes and their associations with mood change outcomes. In addition, we did not report immune response to vaccines, although response has been widely evidenced to be robust. Trajectory of these values could be evaluated in long-term follow-up and may be a source of validation for current study. Although nomogram was developed over a multi-center research protocol, a validation by an independent cohort is required to find predicting values.

## Conclusions

Patients with HT seemed to have increased incidence of mood changes after COVID-19 vaccines. This outcome seems related to changes in the CRP and TSH levels. Baseline serum markers—CRP, TSH, and anti-TPO antibodies—could predict outcome of mood changes and TSH increase after vaccination.

## Data availability statement

The original contributions presented in the study are included in the article/**Supplementary Material**. Further inquiries can be directed to the corresponding authors.

## Ethics statement

The studies involving human participants were reviewed and approved by Second Affiliated Hospital of Shantou University Medical College. The patients/participants provided their written informed consent to participate in this study.

## Author contributions

Prof XW and Dr AZ conceptualized and designed the study, and reviewed and revised the manuscript. Drs YM, NL designed the data collection instruments, carried out the initial analyses, and wrote the first draft of the manuscript. Drs JZ, YJ, YX, YOW, GZ, GJ, YAW, ZZ, GF, SH and CL coordinated and supervised data collection and critically reviewed the manuscript for important intellectual content. Drs YL, SC, XW, PZ, XL, YN, ML and SL collected data and reviewed and revised the manuscript. All authors approved the final manuscript as submitted and agree to be accountable for all aspects of the work. The work reported in the paper has been performed by the authors, unless clearly specified in the text. All authors contributed to the article and approved the submitted version.

## References

[1] Hollowell JG Staehling NW Flanders WD Hannon WH Gunter EW Spencer CA. Serum TSH T(4), and thyroid antibodies in united states population (1988 to 1994): National health and nutrition examination survey (NHANES III). J Clin Endocrinol Metab (2002) 87(2):489–99. doi: 10.1210/jcem.87.2.8182 11836274

[2] Surks MI Ortiz E Daniels GH Sawin CT Col NF Cobin RH. Subclinical thyroid disease: scientific review and guidelines for diagnosis and management. Jama (2004) 291(2):228–38. doi: 10.1001/jama.291.2.228 14722150

[3] Sultanova A Cistjakovs M Gravelsina S Chapenko S Roga S Cunskis E. Association of active human herpesvirus-6 (HHV-6) infection with autoimmune thyroid gland diseases. Clin Microbiol infection Off Publ Eur Soc Clin Microbiol Infect Dis (2017) 23(1):50.e1–5. doi: 10.1016/j.cmi.2016.09.023 27693656

[4] Faustino LC Lombardi A Madrigal-Matute J Owen RP Libutti SK Tomer Y. Interferon-α triggers autoimmune thyroid diseases *via* lysosomal-dependent degradation of thyroglobulin. J Clin Endocrinol Metab (2018) 103(10):3678–87. doi: 10.1210/jc.2018-00541 PMC6179164 30113675

[5] Caselli E D'Accolti M Soffritti I Zatelli MC Rossi R Degli Uberti E. HHV-6A *in vitro* infection of thyrocytes and T cells alters expression of miRNA associated to autoimmune thyroiditis. Virol J (2017) 14(1):3. doi: 10.1186/s12985-016-0672-6 28081700 PMC5234148

[6] Ma Y Liu N Wang Y Zeng J Hu YY Hao W. Immune checkpoint blocking impact and nomogram prediction of COVID-19 inactivated vaccine seroconversion in patients with cancer: a propensity-score matched analysis. J immunotherapy cancer. (2021) 9(11). doi: 10.1136/jitc-2021-003712 PMC8634011 34845005

[7] Rearte A Castelli JM Rearte R Fuentes N Pennini V Pesce M. Effectiveness of rAd26-rAd5, ChAdOx1 nCoV-19, and BBIBP-CorV vaccines for risk of infection with SARS-CoV-2 and death due to COVID-19 in people older than 60 years in Argentina: a test-negative, case-control, and retrospective longitudinal study. Lancet (London England) (2022) 399(10331):1254–64. doi: 10.1016/s0140-6736(22)00011-3 PMC8923678 35303473

[8] Puig-Domingo M Marazuela M Yildiz BO Giustina A. COVID-19 and endocrine and metabolic diseases. an updated statement from European society of endocrinology. Endocrine (2021) 72(2):301–16. doi: 10.1007/s12020-021-02734-w PMC8105151 33963516

[9] Puig-Domingo M Marazuela M Giustina A. COVID-19 and endocrine diseases. a statement from European society of endocrinology. Endocrine (2020) 68(1):2–5. doi: 10.1007/s12020-020-02294-5 32279224 PMC7150529

[10] Passah A Arora S Damle NA Reddy KS Khandelwal D Aggarwal S. Occurrence of subacute thyroiditis following influenza vaccination. Indian J Endocrinol Metab (2018) 22(5):713–4. doi: 10.4103/ijem.IJEM_237_18 PMC6166570 30294587

[11] Paschou SA Karalis V Psaltopoulou T Vasileiou V Charitaki I Bagratuni T. Patients with autoimmune thyroiditis present similar immunological response to COVID-19 BNT162b2 mRNA vaccine with healthy subjects, while vaccination may affect thyroid function: A clinical study. Front endocrinology. (2022) 13:840668. doi: 10.3389/fendo.2022.840668 PMC8902239 35273575

[12] Chen M Zhou W Xu W. Thyroid function analysis in 50 patients with COVID-19: A retrospective study. Thyroid Off J Am Thyroid Assoc (2021) 31(1):8–11. doi: 10.1089/thy.2020.0363 32600165

[13] Lania A Sandri MT Cellini M Mirani M Lavezzi E Mazziotti G. Thyrotoxicosis in patients with COVID-19: THYRCOV study. Eur J Endocrinol (2020) 183(4):381–7. doi: 10.1530/eje-20-0335 PMC9494315 32698147

[14] Siegmann EM Müller HHO Luecke C Philipsen A Kornhuber J Grömer TW. Association of mood changes and anxiety disorders with autoimmune thyroiditis: A systematic review and meta-analysis. JAMA Psychiatry (2018) 75(6):577–84. doi: 10.1001/jamapsychiatry.2018.0190 PMC6137529 29800939

[15] Degner D Haust M Meller J Rüther E Reulbach U. Association between autoimmune thyroiditis and depressive disorder in psychiatric outpatients. Eur Arch Psychiatry Clin Neurosci (2015) 265(1):67–72. doi: 10.1007/s00406-014-0529-1 25193677

[16] Perlis RH Ognyanova K Santillana M Lin J Druckman J Lazer D. Association of major depressive symptoms with endorsement of COVID-19 vaccine misinformation among US adults. JAMA network Open (2022) 5(1):e2145697. doi: 10.1001/jamanetworkopen.2021.45697 35061036 PMC8783266

[17] Hirtz R Föcker M Libuda L Antel J Öztürk D Kiewert C. Increased prevalence of subclinical hypothyroidism and thyroid autoimmunity in depressed adolescents: Results from a clinical cross-sectional study in comparison to general pediatric population. J Clin Psychiatry (2021) 82(2). doi: 10.4088/JCP.20m13511 33989468

[18] van de Ven AC Muntjewerff JW Netea-Maier RT de Vegt F Ross HA Sweep FC. Association between thyroid function, thyroid autoimmunity, and state and trait factors of mood changes. Acta psychiatrica Scandinavica (2012) 126(5):377–84. doi: 10.1111/j.1600-0447.2012.01870.x 22533798

[19] Buganza-Torio E Mitchell N Abraldes JG Thomas L Ma M Bailey RJ. Mood changes in cirrhosis - a retrospective evaluation of prevalence, predictors and development of a screening nomogram. Alimentary Pharmacol &rapeutics (2019) 49(2):194–201. doi: 10.1111/apt.15068 30485460

[20] Austin PC. Balance diagnostics for comparing distribution of baseline covariates between treatment groups in propensity-score matched samples. Stat Med (2009) 28(25):3083–107. doi: 10.1002/sim.3697 PMC3472075 19757444

[21] Brancatella A Ricci D Viola N Sgrò D Santini F Latrofa F. Subacute thyroiditis after sars-COV-2 infection. J Clin Endocrinol Metab (2020) 105(7). doi: 10.1210/clinem/dgaa276 PMC7314004 32436948

[22] Yalcin MM Altinova AE Cavnar B Bolayir B Akturk M Arslan E. Is thyroid autoimmunity itself associated with psychological well-being in euthyroid hashimoto's thyroiditis? Endocrine J (2017) 64(4):425–9. doi: 10.1507/endocrj.EJ16-0418 28260699

[23] Osimo EF Perry BI Cardinal RN Lynall ME Lewis J Kudchadkar A. Inflammatory and cardiometabolic markers at presentation with first episode psychosis and long-term clinical outcomes: A longitudinal study using electronic health records. Brain behavior Immun (2021) 91:117–27. doi: 10.1016/j.bbi.2020.09.011 PMC7773969 32950620

[24] Carty CL Heagerty P Nakayama K McClung EC Lewis J Lum D. Inflammatory response after influenza vaccination in men with and without carotid artery disease. Arteriosclerosis thrombosis Vasc Biol (2006) 26(12):2738–44. doi: 10.1161/01.ATV.0000248534.30057.b5 17023683

[25] McGovern D Williams SP Parsons K Farrah TE Gallacher PJ Miller-Hodges E. Long-term outcomes in elderly patients with ANCA-associated vasculitis. Rheumatol (Oxford England) (2020) 59(5):1076–83. doi: 10.1093/rheumatology/kez388 PMC7671635 31794032

[26] deFilippi C Wasserman S Rosanio S Tiblier E Sperger H Tocchi M. Cardiac troponin T and c-reactive protein for predicting prognosis, coronary atherosclerosis, and cardiomyopathy in patients undergoing long-term hemodialysis. Jama (2003) 290(3):353–9. doi: 10.1001/jama.290.3.353 12865376

