# Supplementary material for: Thyroid function and associated mood changes after COVID-19 vaccines in patients with Hashimoto thyroiditis
Source: Front Immunol. 2023 Apr 6;14:1129746. doi: 10.3389/fimmu.2023.1129746 (PMC10117681; doi:10.3389/fimmu.2023.1129746)
Supplement: Supplementary file 1 [file DataSheet_1.docx]

| **Supplementary Table 1, detailed balance test results in propensity score matching between the vaccine and the control group** | | | | | | | | |
| --- | --- | --- | --- | --- | --- | --- | --- | --- |
| **Covariates** | **Vaccine** | | **Control** | | **SD of the Control** | | **SMD** | |
|  | **unmatched** | **Matched** | **Unmatched** | **Matched** | **Unmatched** | **Matched** | **Unmatched** | **Matched** |
| **Propensity** | 0.74 | 0.63 | 0.55 | 0.62 | 0.22 | 0.18 | 1.18 | 0.04 |
| **Female** | 0.85 | 0.72 | 0.66 | 0.75 | 0.47 | 0.43 | 0.54 | -0.07 |
| **Male** | 0.15 | 0.28 | 0.34 | 0.25 | 0.47 | 0.43 | -0.54 | 0.07 |
| **Age** | 42.34 | 47.39 | 51.84 | 48.69 | 14.91 | 14.01 | -0.68 | -0.09 |
| **TSH** | 293.29 | 291.13 | 292.60 | 292.14 | 119.35 | 119.30 | 0.01 | -0.01 |
| **CRP** | 58.11 | 48.75 | 43.50 | 44.74 | 47.67 | 48.92 | 0.18 | 0.05 |
| **TPO-Ab, IU/mL** | 38.36 | 48.30 | 56.82 | 50.92 | 121.81 | 112.68 | -0.18 | -0.03 |
| **Comorbid with AIID** | 0.04 | 0.08 | 0.09 | 0.07 | 0.29 | 0.25 | -0.29 | 0.09 |
| **Psychiatric history** | 0.18 | 0.22 | 0.24 | 0.22 | 0.42 | 0.42 | -0.14 | < 0.01 |
| **Time since dignosis** | 9.60 | 9.40 | 10.14 | 9.74 | 6.65 | 6.64 | -0.05 | -0.03 |
| SMD,standardized mean difference; SD, standard deviation; AIID, autoimmune inflammatory diseases; BDI-13, Beck Depression Index-13; TSH, thyroid stimulating hormones; CRP, C-reactive proteins. | | | | | | | | |

***Supplementary Material***

**Thyroid function and associated mood changes after COVID-19 vaccines in patients with Hashimoto thyroiditis**

| **Supplementary Table 2. Regression Analysis of TSH increases after vaccination** | | | | | | |
| --- | --- | --- | --- | --- | --- | --- |
|  |  | **TSH changes** | |  |  |  |
| **Factors** | | **Increased**  **(N = 291, 10.5%)** | **Not increased**  **(N = 2474, 89.5%)** | ***p* (Univariate)** | ***p* (Multivariate)** | **Odds Ratio** |
| **Gender** | **Male** | 51 (17.5) | 364 (14.7) | 0.20 | — | — |
|  | **Female** | 240 (82.5) | 2110 (85.3) |  |  |  |
| **Age, years** | | 41.51 (13.98) | 42.44 (13.99) | 0.28 | — | — |
| **Time since diagnosis** | | 9.92 (11.29) | 9.56 (10.91) | 0.60 | — | — |
| **TSH, μIU/dL** | | 335.82 (119.55) | 288.28 (109.68) | < 0.01 | < 0.01 | 1.003 |
| **CRP, μg/dL** | | 919.90 (819.81) | 541.27 (776.86) | < 0.01 | < 0.01 | 1.002 |
| **IL-6, pg/mL** | | 201.89 (135.10) | 140.51 (96.94) | < 0.01 | < 0.01 | 1.005 |
| **Anti-TG antibody, IU/mL** | | 66.45 (117.16) | 95.19(395.28) | 0.23 |  |  |
| **Anti-TPO antibody, IU/mL** | | 88.67 (175.08) | 32.44 (85.86) | < 0.01 | < 0.01 | 1.002 |
| **Comorbidity with other AIID** | **Yes** | 22 | 82 | < 0.01 | < 0.01 | 4.75 |
|  | **No** | 269 | 2392 |  |  |  |
| **BDI values** | | 8.38 (6.13) | 8.54 (5.04) | 0.62 | — | — |
| **Psychiatric disease history** | **Yes** | 53 (18.2) | 446 (18.0) | 0.94 | — | — |
|  | **No** | 238 (81.8) | 2028 (82.0) |  |  |  |
| SD, standard deviation; AIID, autoimmune inflammatory diseases; BDI-13, Beck Depression Index-13; TSH, thyroid stimulating hormones; CRP, C-reactive proteins. | | | | | | |


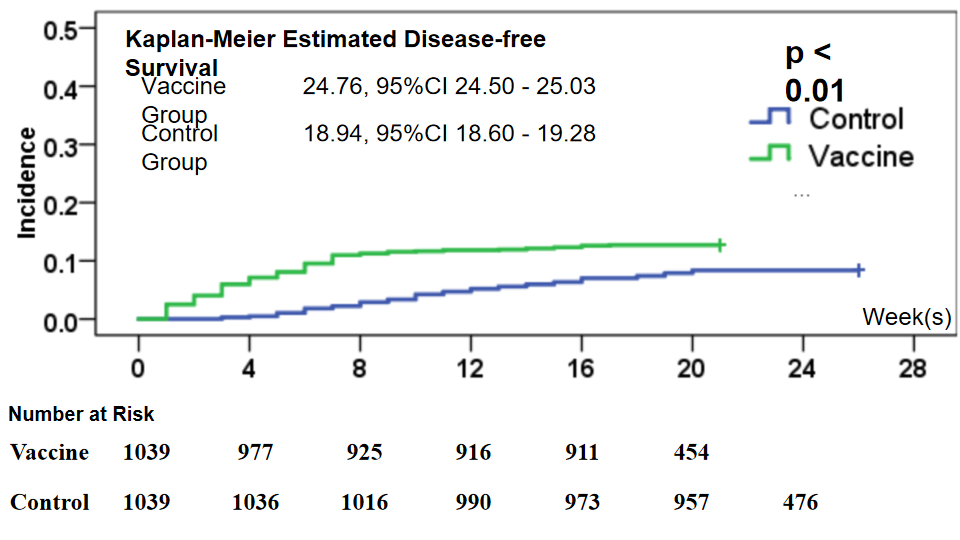


**Supplementary Figure 1**

Kaplan-Meier estimated disease-free survival curve in the vaccine group and control group.


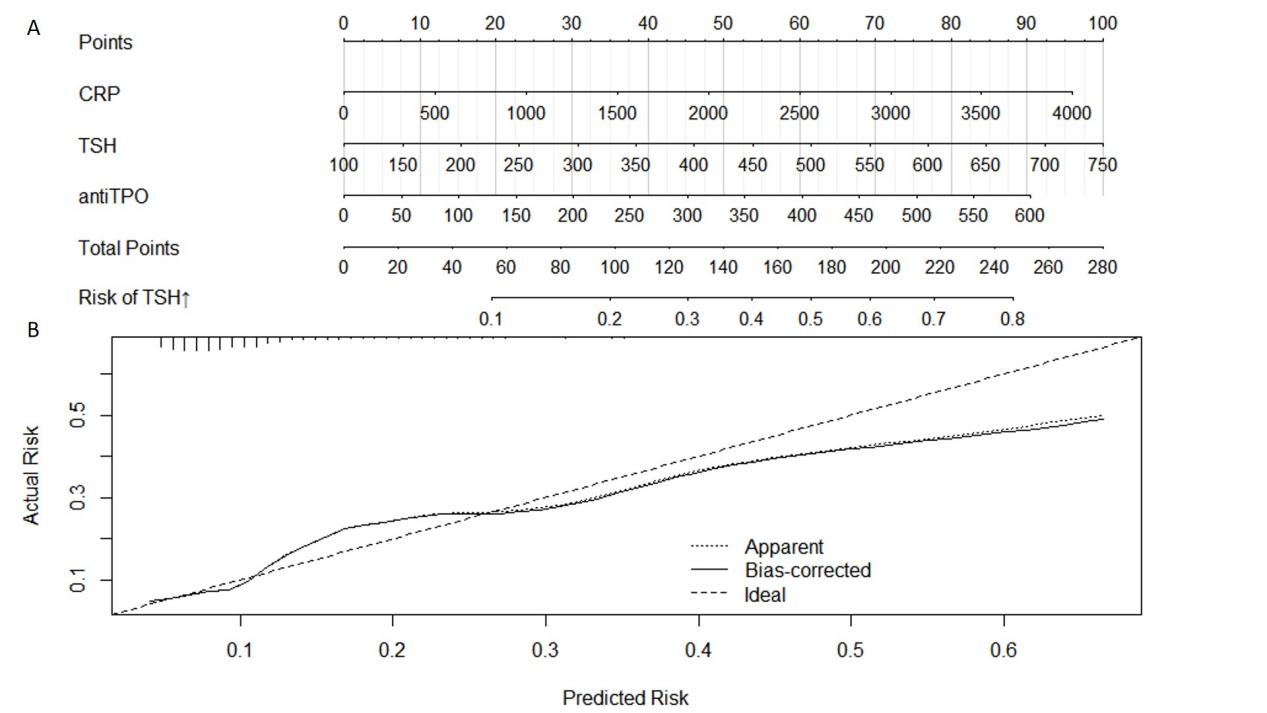


**Supplementary Figure 2**

A, Nomogram predicting the risk of TSH increase (> 200 μIU/dL) 2 months after vaccination. B, calibration curve showing agreement between predicted and actual rate of TSH increase.
